# S08 Do results from physical activity questionnaires (IPAQ, GPAQ, EHIS and Eurobarometer) and accelerometer tell same story - results from 18 countries in the EUPASMOS project

**DOI:** 10.1093/eurpub/ckac093.039

**Published:** 2022-08-29

**Authors:** Tommi Vasankari, Paulo Rocha, Ellen de Hollander

**Affiliations:** 1 UKK Institute for Health Promotion Research, Tampere, Finland; 2 Portuguese Institute of Sport and Youth, Lisbon, Portugal; 3 National Institute for Public Health and the Environment, Bilthoven, The Netherlands

**Keywords:** accelerometer, breiks, physical fitness, sedentary behaviour, questionnaire

## Abstract

Physical activity (PA) is associated with reduced risk of many noncommunicable diseases while a high volume of sedentary behavior (SB) is also considered to increase the risk of these diseases. Accurate measurement of both PA and SB is crucial for estimating these risk factors, and accelerometers together with standardized questionnaires are suggested a feasible way to measure these behaviors. However, collecting raw acceleration data of PA, SB and sleep in a standardized and similar way on a large scale has not been done before. In the European Union Physical Activity and Sport Monitoring System project (EUPASMOS), this has been done for the first time in 18 EU Member States.

